# A Time-Series Approach for Machine Learning-Based Patient-Specific Quality Assurance of Radiosurgery Plans

**DOI:** 10.3390/bioengineering12080897

**Published:** 2025-08-21

**Authors:** Simone Buzzi, Pietro Mancosu, Andrea Bresolin, Pasqualina Gallo, Francesco La Fauci, Francesca Lobefalo, Lucia Paganini, Marco Pelizzoli, Giacomo Reggiori, Ciro Franzese, Stefano Tomatis, Marta Scorsetti, Cristina Lenardi, Nicola Lambri

**Affiliations:** 1 Radiotherapy and Radiosurgery Department, IRCCS Humanitas Research Hospital, Via Manzoni 56, Rozzano, 20089 Milan, Italy; simonebuzzi13@gmail.com (S.B.); andrea.bresolin@humanitas.it (A.B.); pasqualina.gallo@humanitas.it (P.G.); francesco.lafauci@humanitas.it (F.L.F.); francesca.lobefalo@humanitas.it (F.L.); lucia.paganini@cancercenter.humanitas.it (L.P.); marco.pelizzoli@humanitas.it (M.P.); giacomo.reggiori@humanitas.it (G.R.); stefano.tomatis@cancercenter.humanitas.it (S.T.); nicola.lambri@unimi.it (N.L.); 2 Dipartimento di Fisica “Aldo Pontremoli”, Università degli Studi di Milano, 20133 Milan, Italy; cristina.lenardi@unimi.it; 3 Department of Biomedical Sciences, Humanitas University, Via Rita Levi Montalcini 4, Pieve Emanuele, 20072 Milan, Italy; ciro.franzese@hunimed.eu (C.F.); marta.scorsetti@hunimed.eu (M.S.); 4 Milano Division, National Institute for Nuclear Physics, 20133 Milan, Italy; 5 Scuola di Specializzazione di Fisica Medica, Università degli Studi di Milano, 20133 Milan, Italy

**Keywords:** machine learning, patient-specific QA, stereotactic radiosurgery, HyperArc, radiotherapy

## Abstract

Stereotactic radiosurgery (SRS) for multiple brain metastases can be delivered with a single isocenter and non-coplanar arcs, achieving highly conformal dose distributions at the cost of extreme modulation of treatment machine parameters. As a result, SRS plans are at a higher risk of patient-specific quality assurance (PSQA) failure compared to standard treatments. This study aimed to develop a machine-learning (ML) model to predict the PSQA outcome (gamma passing rate, GPR) of SRS plans. Five hundred and ninety-two consecutive patients treated between 2020 and 2024 were selected. GPR analyses were performed using a 3%/1 mm criterion and a 95% action limit for each arc. Fifteen plan complexity metrics were used as input features to predict the GPR of an arc. A stratified and a time-series approach were employed to split the data into training (1555 arcs), validation (389 arcs), and test (486 arcs) sets. The ML model achieved a mean absolute error of 2.6% on the test set, with a 0.83% median residual value (measured/predicted). Lower values of the measured GPR tended to be overestimated. Sensitivity and specificity were 93% and 56%, respectively. ML models for virtual QA of SRS can be integrated into clinical practice, facilitating more efficient PSQA approaches.

## 1. Introduction

Intensity-modulated radiotherapy (IMRT) and volumetric modulated arc therapy (VMAT) are inverse treatment planning techniques widely used for their ability to deliver highly conformal dose distributions, capable of maximizing tumor targeting while sparing healthy tissue [1]. This is especially true for stereotactic radiosurgery (SRS), which involves the delivery of highly conformal dose distributions with steep dose gradients to brain lesions, administered in a single or a few high-dose fractions. For this reason, measurement-based patient-specific quality assurance (PSQA) is mandatory to verify that SRS treatments can be delivered as intended [2,3].

Typically, the measured and calculated dose distributions are compared both for dose difference and physical distance using the gamma agreement index (γ) [4]. The PSQA result is summarized by the gamma passing rate (GPR), i.e., the percentage of points where γ ≤ 1. The AAPM TG-218 recommends, for general IMRT and VMAT treatments, a 3%/2 mm gamma criterion, using global normalization, with a 10% dose threshold and a 90% action limit [5]. The same report recommends stricter, albeit unspecified, criteria for SRS.

Measurement-based PSQA is inherently a time-consuming and resource-intensive process. In addition, PSQA failures can lead to increased workload and delays in patient treatment due to the need for replanning. In the search for alternatives, several studies have explored the use of complexity metrics to establish a relationship with PSQA outcomes [6,7,8,9,10]. Plan complexity attempts to quantify the uncertainties related to approximations in the dose calculation algorithms of a treatment planning system (TPS) and the mechanical limitations of a linear accelerator (linac), which could affect the results of PSQA. However, no final consensus has been reached, as results show strong correlations only in some cases, while also being dependent on the QA device, treatment technique, and linac used [10,11]. More recently, machine-learning (ML) [12,13,14,15,16,17,18] and deep-learning (DL) models [19,20,21] have been investigated to predict PSQA outcomes from complexity metrics. Overall, these studies varied greatly in terms of treatment sites, analysis methods, input features, and algorithms used. Moreover, specific applications to SRS treatments remain unexplored.

In this study, we considered a dataset of multiple target single-isocenter SRS plans, which are more subject to PSQA failures than standard treatments due to their inherent higher complexity. A virtual QA method specific to these cases could potentially reduce a department’s QA workload by identifying plans at risk of failure before measurement. To this aim, a tree-based ML model was trained and tested employing a time-series split to assess the model’s applicability under similar conditions to clinical practice.

## 2. Materials and Methods

### 2.1. Dataset

Five hundred ninety-two (592) consecutive SRS patients treated between December 2020 and December 2024 were selected from the internal database of our Institute, for a total of 2430 arcs. The RT plans were optimized using HyperArc (v15; Varian Medical System, Palo Alto, CA, USA), a VMAT optimization algorithm specifically developed for treating multiple brain lesions simultaneously, using a single isocenter for radiation delivery, achieving dose delivery accuracy and conformity through the use of non-coplanar arcs and extreme modulation of MLC leaf positions [22]. A Varian Edge linac was employed, equipped with a Varian High Definition 120 MLC (Varian Medical System, Palo Alto, CA, USA). The MLC features, for each bank, 32 × 2.5 mm-wide inner leaves and 28 × 5 mm-wide outer leaves.

PSQA measurements were performed using an Electronic Portal Imaging Device (EPID) mounted on the linac. The EPID was a Varian PortalVision aS1200 Imager (Varian Medical System, Palo Alto, CA, USA), which features an amorphous silicon (a-Si) detector panel with a size of 43 × 43 cm^2^, corresponding to a pixel matrix of 1280 × 1280 with a resolution of 0.336 mm. The gamma analyses were performed with Portal Dosimetry (v15; Varian Medical System, Palo Alto, CA, USA). This software computes the GPR by comparing the dose distribution calculated by the TPS to the integral delivered dose measured with the EPID. The conversion between dose distribution and portal dose image was performed by the Portal Dosimetry Image Prediction (v15; Varian Medical System, Palo Alto, CA, USA) algorithm. The parameters used to compute the GPR were 3%/1 mm, with a 10% dose threshold and global normalization in absolute dose. These criteria were based on the department’s QA program for SRS treatments [23]. The monthly quality assurance procedure applied to the portal imager consisted of dark field and flood field checks, followed by normalization using a 10 × 10 cm^2^ calibration field.

As input features, originally two static parameters, six dynamic parameters, and ten complexity metrics were employed, as reported in Table 1. These had been extracted from DICOM RT plan files by means of a software program written in MATLAB (R2021b) [24]. To mitigate the impact of multicollinearity on feature importance algorithms, Spearman’s correlation coefficients (r_s_) between features were calculated. Hierarchical clustering with Ward’s linkage was then applied to a distance matrix derived as 1 − |r_s_|. A stringent distance threshold of 0.1 was applied to the resulting dendrogram. This threshold, interpreted on the scale of Ward’s linkage criterion, was selected with the objective of grouping features with an absolute Spearman’s correlation (|r_s_|) of 0.9 or higher. From the clusters formed, one representative feature was retained per cluster, leading to a selection of fifteen features. The selected features are reported in italics in Table 1.

### 2.2. Model Selection

A random forest model was trained to predict the GPR of an arc based on its complexity. During early experiments, the model exhibited difficulty in predicting low GPR values, which were underrepresented in the training set. To address this, sample weights were assigned to each training instance, prioritizing these low GPR values according to the following relation:

(1) $$w_{i}=\left(100.1-{GPR}_{i}\right),\;where\;{GPR}_{i}\;\in \;training\;set.$$

**Figure 1:**
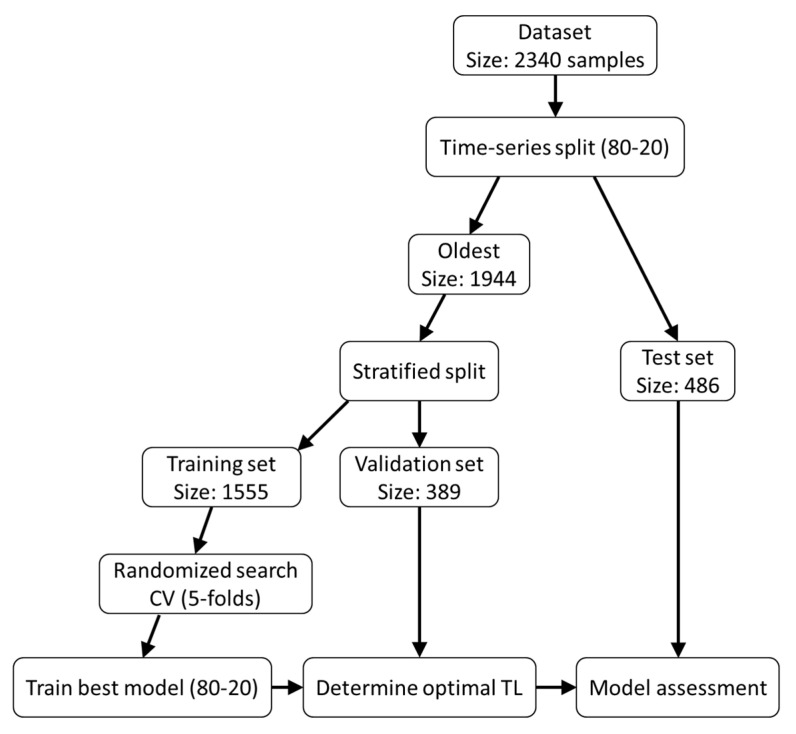
Pipeline for model selection and assessment. Abbreviations: CV = cross-validation; TL = threshold limit.

The ML pipeline implemented in this study aimed to mimic real-world scenarios where models are evaluated on future data. The approach is outlined in Figure 1 and consists of the following steps:

1. The original dataset was first divided using a time-series approach, with the most recent 20% of data assigned to the test set. This proportion was selected to represent approximately 6 months of clinical PSQA workload, enabling a realistic assessment of the model’s prospective performance. The remaining 80%—consisting of the oldest data—was further split into training and validation sets using a stratified approach on GPR with an 80-20 ratio. As a result, the training, validation, and test sets contained 1555, 389, and 486 arcs, respectively.
2. A randomized search with 5-fold cross-validation was performed to select the optimal combination of hyperparameters using 1000 iterations. The mean absolute error (MAE) was used as the evaluation metric, averaged across the cross-validation folds. Features were rescaled using percentile statistics as follows, which are robust to outliers:
  (2) $$f_{scaled}=\frac{f\;-\;f_{50\%}}{f_{75\%}\;-\;f_{25\%}}$$
  To prevent data leakage, this scaling operation was performed independently within each training fold.
3. Using the optimal combination of hyperparameters (reported in the Supplementary Material, Table S1), the model was retrained on the whole training set.
4. The validation set was used for determining an optimal threshold limit (TL) to assess the classification performance of the model on the test set, which contained the most recent data.

### 2.3. Model Assessment

The performance of the regression model on the test set was evaluated using the R^2^, MAE, and absolute error statistics. R^2^ measures the proportion of variance in the GPR explained by the model, where the best possible score is 1 and a value of 0 denotes a model that always predicts the average value of GPR.

Arcs were divided into a positive class, with measured GPR < 95%, and a negative class, with measured GPR ≥ 95%. The decision to use a 95% action limit, recommended as the tolerance limit in AAPM guidelines, was based on the fact that AAPM’s recommendations are for RT in general, while stricter, albeit unspecified, criteria are recommended for SRS [5].

The classification performance of the model was assessed based on receiver-operating-characteristic (ROC) and precision–recall curves. These curves were obtained by computing the sensitivity/specificity and precision/recall for all possible action limits on the GPR predicted by the model. In this study, sensitivity (recall) and specificity measured the ability of the model to correctly identify “fail” (positive class) and “pass” (negative class) arcs, while precision measured the fraction of true “fails” identified among the predicted failures. These metrics were computed as follows:

(3) $$sensitivity=\frac{\#true\;fail\;}{\#true\;fail\;+\;\#false\;pass},$$

(4) $$specificity=\frac{\#true\;pass\;}{\#true\;pass+\#false\;fail},$$

(5) $$precision=\frac{\#true\;fail\;}{\#true\;fail+\#false\;fail}$$

An optimal TL was determined from the validation set as a threshold ensuring at least 90% sensitivity and 50% specificity. Achieving 100% sensitivity would require a threshold that produces an unacceptably high false-positive rate, making the model impractical for routine use. This threshold was then applied to the test set to compute sensitivity, specificity, and precision.

### 2.4. Interpretability

Partial dependence plots (PDPs) were used as a post hoc method to explain the model output and interpret feature importance. A PDP shows the global relation between a feature and the target variable, marginalizing over the values of the other variables.

## 3. Results

The Median GPR of the full dataset was 98%. The GPR statistics for different ranges are reported in Table 2. Approximately 75% of the arcs (1828) had a GPR ≥ 95%.

The correlation heatmap and dendrogram between feature pairs are reported in Figures S1 and S2 of the Supplementary Material. A strong correlation (r_s_ > 0.9) was found between the Q1 MLCGap and Median MLCGap, which describe similar quantities. Strong anti-correlation (r_s_ < −0.9) was observed between three pairs: BM and MCS, Median MLCGap and SAS10, and Q1 MLCGap and SAS10.

The model assessment is summarized in Table 3. We found similar MAEs obtained with cross-validation (2.5% ± 0.1%) and on the test set (2.6%). While 95% of data points fall within a 7.5% absolute error, the 98th percentile corresponds to a 10% absolute error. The R^2^ value was found to be 0.27, while the ROC and precision–recall curve analyses resulted in an AUC of 0.85 and AP of 0.67. In particular, both were above the baseline values of a dummy classifier. Using the 97% TL obtained from the validation set, the sensitivity, specificity, and precision on the test set were 93%, 56%, and 50%, respectively.

**Figure 2:**
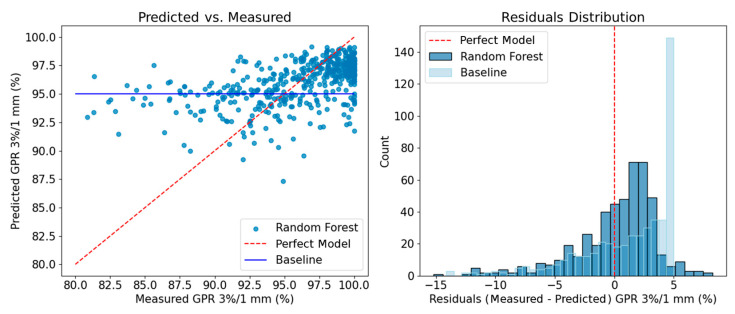
Scatter plot (**left**) and residual distribution (**right**) of the measured and predicted GPRs. The dashed lines represent a perfect regression model, while the solid lines denote a baseline model always predicting the average GPR.

Figure 2 shows the scatter plot and residual distribution of the model on the test set. The model overestimated the measured GPR below 95%, yielding mean and median errors of −4.2% and −3.8%, respectively, for GPR values below the 1st quartile (<95.1%). The median residual was 0.85% with the 1st and 3rd quartiles of [−1.4, 2.3]%. The maximum residual was near 15%.

**Figure 3:**
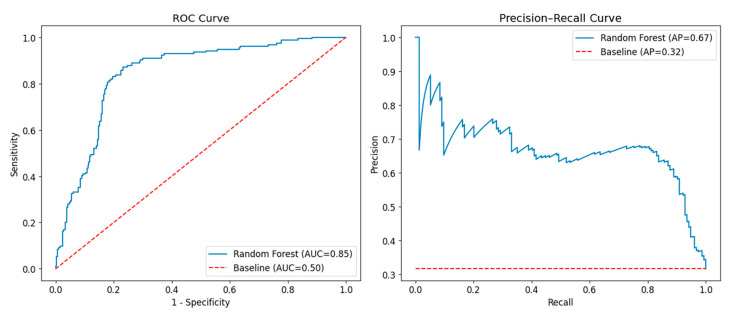
ROC and precision–recall curves on the test set. The dashed lines denote a dummy classifier always predicting the most frequent class (i.e., “pass”). AP = average precision; ROC: receiver operating characteristic.

Figure 3 shows the ROC and precision–recall curves obtained on the test set. Considering the clinical action limit, 95%, for the predicted GPR as TL, the model sensitivity and specificity were 86% and 53%, respectively.

**Figure 4:**
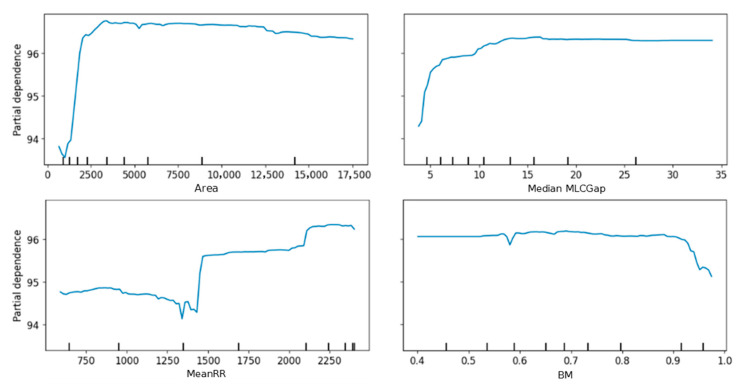
PDP plots on the test set for the Area, Median MLCGap, MeanRR, and BM.

The PDPs for the Area, Median MLCGap, MeanRR, and BM, based on the test set, are shown in Figure 4. These are the main features showing a non-trivial dependence, while the remaining features are reported in Figure S3 of the Supplementary Material. The Area and Median MLCGap show similar patterns; smaller values are associated with lower predictions, while at increasing values, they do not show any influence on the outcome. The MeanRR exhibits a decrease in predicted values at low ranges, followed by two sharp increases as its values rise. BM shows a different trend, as it only begins to affect predictions significantly at higher values.

## 4. Discussion

In this study, an ML model was developed to investigate the potential of reducing the PSQA workload of SRS plans treating multiple brain metastases with single-isocenter non-coplanar fields. These cases are more subject to PSQA failures than standard treatments due to their inherent higher complexity. The dataset comprised 2430 SRS arcs from 592 consecutive patients. Data split utilized a hybrid approach combining time-series and stratified sampling, reflecting clinical scenarios where models are deployed on future data, as described by Chan et al. [29]. Using a 3%/1 mm criterion and a 95% action limit, the model achieved a 2.6% test set MAE, with a 0.27 R^2^ and 0.85 ROC-AUC, respectively.

The Area, Median MLCGap, MeanRR, and BM proved the most impactful features according to PDPs. The first three in particular displayed a threshold-like behavior. The lower predictions observed for smaller values of the Area and Median MLCGap are likely associated with increased uncertainty in small-field dose calculations, arising from limitations in modeling radiation transmitted at the tip of the MLC leaves. A similar explanation may account for the behavior observed for BM > 0.9, which indicates beams composed of many small apertures that are spatially separated from each other. In contrast, MeanRR exhibited an opposite relationship to what might be expected, possibly suggesting the presence of interaction effects that are not captured by the PDP.

As alternatives to measurement-based PSQA, a few ML and DL methods have been explored in recent years [29,30,31]. Table S2 in the Supplementary Material summarizes some of the relevant studies and compares their main findings with the present work. Lam et al. [12] and Zhu et al. [18] presented the application of complexity metrics-based models for PSQA across various treatment sites, both employing the 2%/2 mm criterion. Although they reported a lower MAE, approximately 1%, the former’s dataset consisted exclusively of 182 IMRT plans with a measured GPR > 90%, and the latter only included 2% plans with GPR < 90%, out of 213. Kusunoki et al. [16] used a 2%/2 mm criterion and developed a series of models utilizing 15 features to analyze a dataset of 356 VMAT plans for the head and neck. With a 99% lower control limit for prediction, they obtained 100% sensitivity and 75% specificity, but the measured GPR range was above 95.2%. Li et al. [13] analyzed 303 VMAT plans from gynecological and head and neck tumor locations and achieved a test MAE of 2.39%, with 100% sensitivity and 71% specificity, although only 2.1% of the plans did not meet the 3%/2 mm criterion with a 90% action limit. Hirashima et al. used the same criterion and action limit to obtain an MAE of 3.1%, sensitivity of 64%, and specificity of 82%, on a dataset of 1255 VMAT plans from various treatment locations [14].

Among those including a higher number of outliers, Wall and Fontenot [15] analyzed 500 VMAT plans from various tumor locations to compare various ML models, with the best model achieving an MAE < 4% at 3%/3 mm. Han et al. [32] developed DL models using 13 plan metrics and a dataset of 201 VMAT plans from pelvis and head and neck tumor locations, with 10% of the plans failing to meet the 3%/2 mm criterion with a 90% action limit. They achieved 87.5% sensitivity and 71.7% specificity. Finally, in our previous study, we analyzed the largest single-institute dataset of VMAT plans to date [17], consisting of 5522 VMAT plans from various treatment sites. The GPR was computed with a 3%/1 mm criterion, and a 95% action limit with a 10% threshold was used. Employing the same set of features as this study, a test MAE of 2.3% was achieved. However, the data split did not consider the temporal dependence, and less than 17% of the arcs had a measured GPR < 95%.

Only Noblet et al. [33], to the best of our knowledge, have explicitly reported a data-splitting approach similar to ours. Their method involved an initial time-series split to separate the most recent data, followed by a random split of the remaining dataset. Their nine classifiers on a dataset of 1767 VMAT arcs from various treatment sites achieved an ROC-AUC of 0.88, with 52% sensitivity and 92% specificity.

By adopting a 97% TL on the test set, our model achieved a sensitivity of 93% and a specificity of 56%. Sensitivity was prioritized over specificity, as missing a true failure carries a greater potential risk than investigating a false alarm. Such a configuration supports the model’s potential role as a complementary tool to our department’s PSQA program, in which SRS plans are always verified. The model could serve as a pre-screening method to anticipate failing cases and reduce the workload burden of repetitive replanning and measurement. In particular, the flagged “fail” cases could be reoptimized with automatable procedures, which reduce plan complexity and improve the GPR [34]. Assuming 25% of failing cases, as observed in this study’s dataset, and that one reoptimization is performed in 10 min while replanning and measurement take 60 min, the maximum expected workload reduction is 56%. The detailed calculation is reported in Section S1 of the Supplementary Material.

According to our department’s PSQA program, SRS plans are verified on an arc basis. This ensures that potential delivery errors in single arcs are not obscured by the superposition of the other beam doses. Due to the extreme modulation of SRS arcs, if an arc fails the PSQA, then the plan is reoptimized. The model performance was derived from the GPR of each arc to reflect this practice, and the metrics reported in this work were used to obtain a global assessment. The achieved level of sensitivity implies that this method is still immature to substitute the conventional PSQA process. Nonetheless, such a model could serve as an additional verification step without changing PSQA procedures.

Although the model was evaluated using a time-series split to assess prospective performance, changes over time in radiotherapy technology, patient characteristics, and clinical practices may still degrade its accuracy. Therefore, the model should be continuously monitored—e.g., by periodically evaluating its performance on a representative set of test plans and checking for prediction drift—and updated as needed to reflect alignment with current clinical practice [35].

It should be noted that EPIDs are perpendicular composite methods, in which the integrated image can mask certain delivery errors. Moreover, EPIDs are not ideal absolute dosimeters and require careful calibration due to their reduced thickness compared with the photon build-up region. A further potential limitation in non-coplanar arcs is that EPID-based PSQA does not fully represent the actual treatment delivery, as it does not account for couch rotation, unlike true composite methods.

Notably, studies including outliers have shown a tendency to overestimate low GPR values. This limitation was also observed in the current work, leading to a low R^2^ score. Several strategies could be explored to improve model performance: increasing the number of samples with low GPR (e.g., by creating fictitious plans with extremely high complexity—although this may reduce the representativeness of the clinical dataset) and expanding the feature set by incorporating dosiomics features [14,32] or metrics derived from linac and EPID QA results. Nonetheless, ML models could provide indicators to anticipate potential failures and be integrated into clinical practice as support tools for an optimized PSQA program.

## 5. Conclusions

A hybrid stratified and time-series approach was applied to train an ML model for predicting PSQA outcomes in highly complex multi-target SRS treatments. The results suggest that plan parameters and complexity metrics provide valuable insights, offering promising potential for training models as supplementary tools for virtual QA, particularly in facilitating more efficient PSQA approaches.

## Figures and Tables

**Table 1:** Input features used for model training. In italics are the fifteen features selected with hierarchical clustering.

#	Name	Description
1	*Area*	Field aperture area (mm^2^)
2	*MUOverDosePerFraction*	Monitor units normalized to the prescribed dose per fraction (MU/Gy)
3	*MeanMLCSpeed*	Mean speed of all in-field leaves (cm/s)
4	*MLCSpeedModulation*	Sum of MLC speed variations divided by total leaf travel (cm/s mm^−1^)
5	*MeanRR*	Mean dose rate (MU/min)
6	*RRModulation*	Total dose rate variation divided by arc length (MU/min deg^−1^)
7	*MeanGS*	Mean gantry speed (deg/s)
8	*GSModulation*	Total gantry speed variation divided by arc length (deg/s deg^−1^)
9	Q1 MLCGap	First quartile of MLC gap size distribution (mm)
10	*Median MLCGap*	Median of MLC gap size distribution (mm)
11	SAS10 [25]	Small aperture score: the fraction of MLC gap sizes < 10 mm
12	*MeanTGI* [9]	Mean tongue and groove index: irregularity in beam aperture shapes
13	MCS [6]	Modulation complexity score: a combination of aperture area variability (AAV) and leaf sequence variability (LSV)
14	*MITotal* [26]	Modulation index total: combines MLC dynamics, gantry speed variability, and dose rate variability
15	*BI* [27]	Beam irregularity: measures the non-circularity of the MLC aperture
16	*BM* [27]	Beam modulation: indicates to what extent the beam is delivered through small apertures
17	*EdgeMetric* [28]	Ratio of MLC side length to aperture area (mm^−1^)
18	*LT/AL* [7]	Average leaf travel distance divided by the arc length (mm/deg)

**Table 2:** Distribution of measured GPR for the full set. Abbreviations: GPR = gamma passing rate; Q1 = 1st quartile; and Q3 = 3rd quartile.

Interval (%)	Median GPR (%)	Q1–Q3 (%)	Number of Arcs
[95, 100]	99.1	97.7–99.8	1828
[90, 95)	93.1	91.8–94.1	400
[85, 90)	88.1	86.9–89.1	151
[80, 85)	83.1	82.1–84.4	51
Full set	98.3	95.1–99.6	2430

**Table 3:** Model assessment. Abbreviations: AbsErr = absolute error; MAE = mean absolute error; ROC-AUC = receiver operating characteristic area under curve; and AP = average precision.

Metric	Value
Cross-validation MAE	2.5% ± 0.1%
Test MAE	2.6%
% arcs with AbsErr ≤ 3%, 5%, 10%	70%, 88%, 98%
75th, 90th, 95th, and 98th percentile of AbsErr	3.3%, 5.5%, 7.5%, 10%
R^2^	0.27 (baseline 0) ^1^
ROC-AUC	0.85 (baseline 0.50)
AP	0.67 (baseline 0.32) ^2^
Sensitivity (97%TL)	93%
Specificity (97% TL)	56%
Precision (97%TL)	50%

^1^ The baseline R^2^ = 0 denotes a model that always predicts the average GPR. ^2^ The baseline AP = 0.32 indicates a model that always predicts the positive class (“fail”).

## Data Availability

The data presented in this study are available on request from the corresponding author.

## Supplementary Materials

The following supporting information can be downloaded at https://www.mdpi.com/article/10.3390/bioengineering12080897/s1: Table S1: Parameters searched in the randomized cross-validation and the resulting best combination; Table S2: Summary of recent studies which investigated the use of ML and DL models for PSQA; Figure S1: Correlation matrix of plan parameters and complexity metrics; Figure S2: Dendrogram of the hierarchical clustering on the Spearman rank–order correlations; Figure S3: PDP plots on the test set for the features showing a trivial dependence.

## Abbreviations

The following abbreviations are used in this manuscript:

AAPM	American Association of Physicists in Medicine
AUC	Area under the curve
DL	Deep learning
EPID	Electronic portal imaging device
GPR	Gamma passing rate
IMRT	Intensity-modulated radiotherapy
Linac	Linear accelerator
MAE	Mean absolute error
ML	Machine learning
PDP	Partial dependence plot
PSQA	Patient-specific quality assurance
ROC	Receiver operating characteristic
SRS	Stereotactic radiosurgery
TL	Threshold limit
TPS	Treatment planning system
VMAT	Volumetric modulated arc therapy
