# Supplementary material for: A Time-Series Approach for Machine Learning-Based Patient-Specific Quality Assurance of Radiosurgery Plans
Source: Bioengineering (Basel). 2025 Aug 21;12(8):897. doi: 10.3390/bioengineering12080897 (PMC12383453; doi:10.3390/bioengineering12080897)
Supplement: Supplementary file 1 [file bioengineering-12-00897-s001.zip › bioengineering-3817419-supplementary.pdf]

Supplementary Material

Table S1: Parameters searched in the randomized cross-validation and the resulting best combination.

Parameter	Values/Range	Best
Number of estimators	Random integer (100-1000)	664
Loss	[linear, square]	square
Base estimator max depth	Random integer (1-20)	18
Base estimator min samples split	Random integer (2-8)	3
Base estimator min samples leaf	Random integer (2-8)	2
Base estimator max features	[sqrt, None]	None

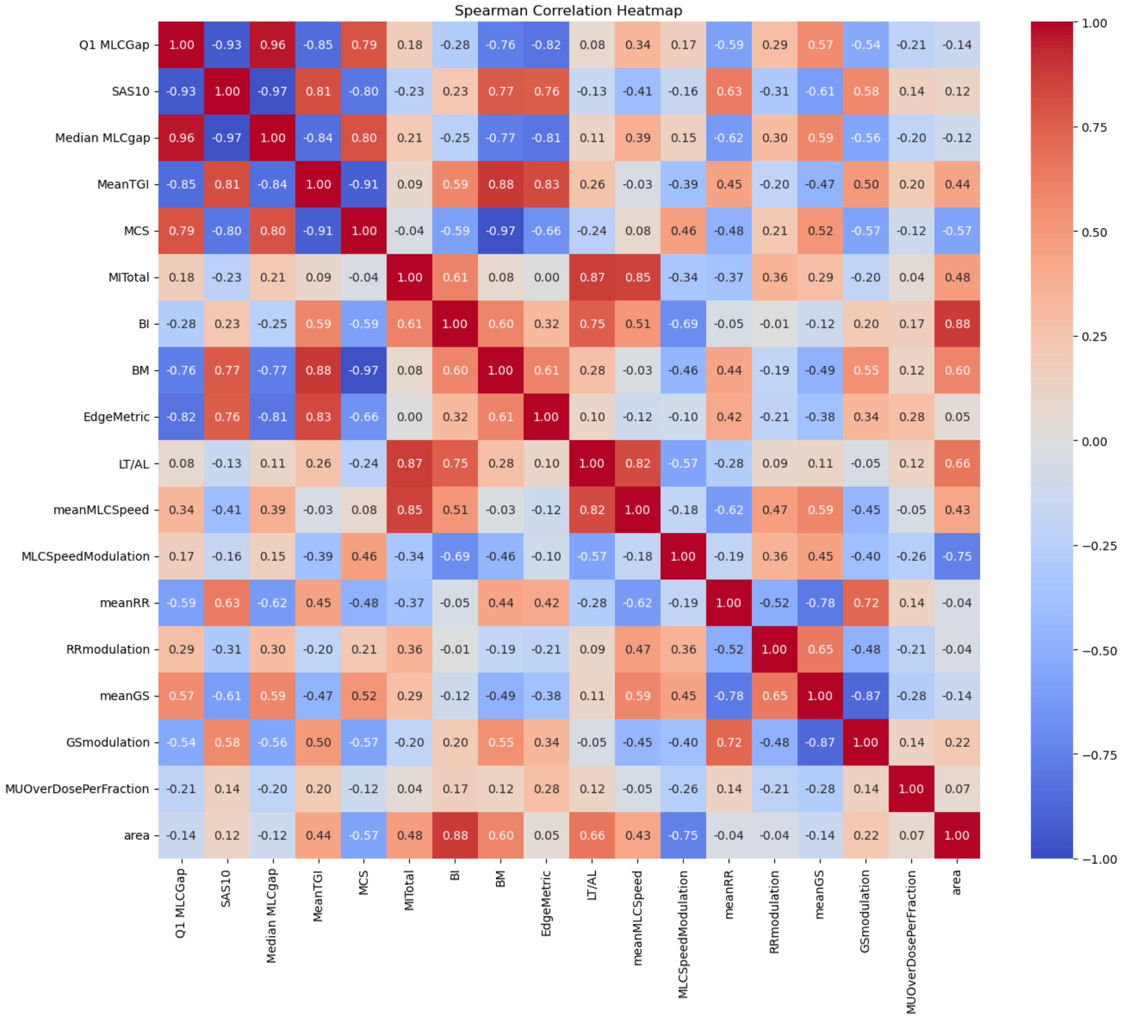

Figure S1: Correlation matrix of plan parameters and complexity metrics.

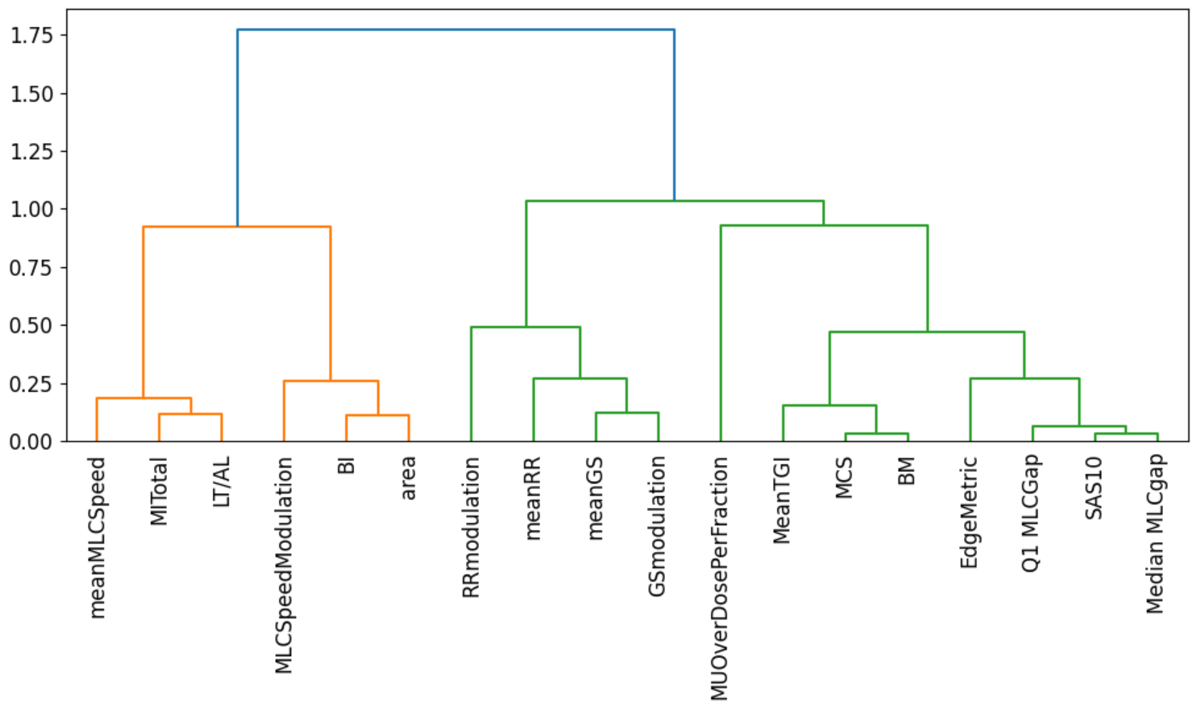

Figure S2: Dendrogram of the hierarchical clustering on the Spearman rank-order correlations.

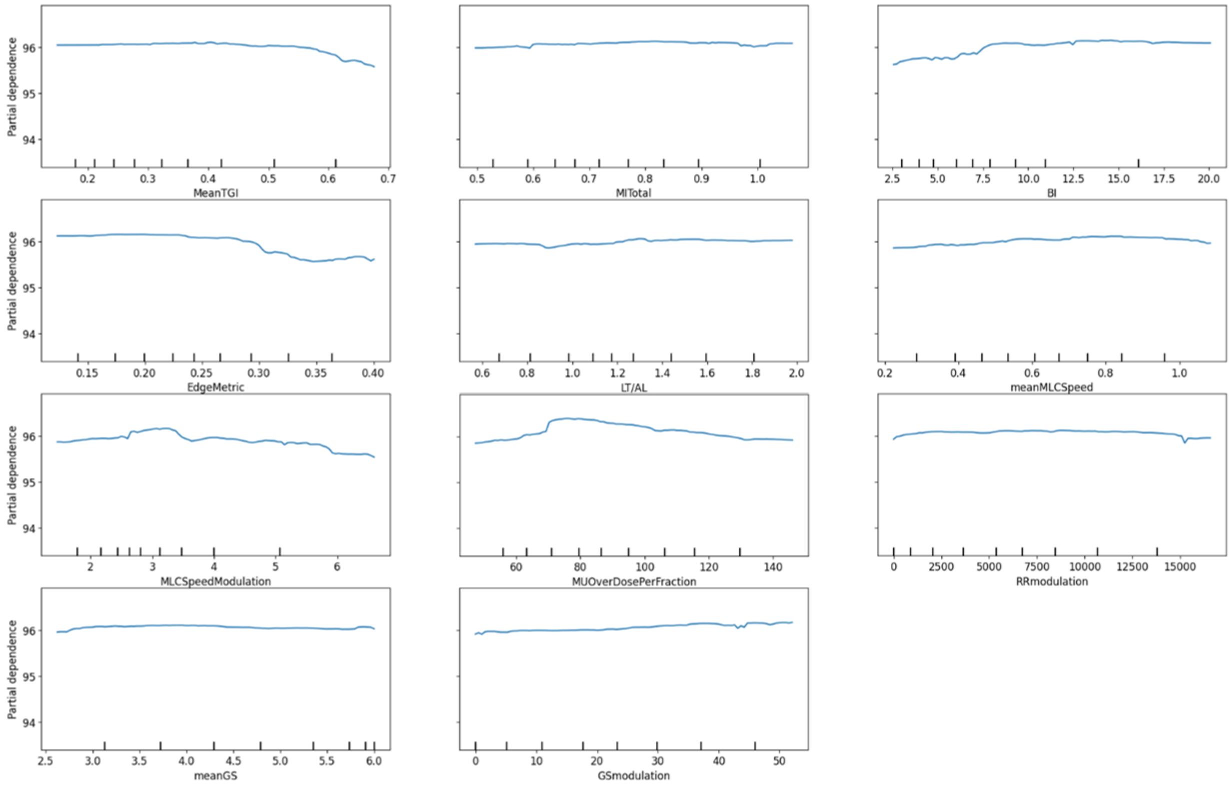

Figure S3: PDP plots on the test set for the features showing a trivial dependence.

Table S2: Summary of recent studies which investigated the use of ML and DL models for PSQA.

Authors	Dataset	Treatment Site	Treatment Unit	Dose Calculation	QA System	γ Criteria	GPR Distribution	Features	ML Model	Results
Lam et al. [1] (2019)	1497 IMRT fields	Multiple	TrueBeam, Trilogy<br><br>6 MV, 10 MV<br><br>Millennium 120 MLC	Algorithm N/A<br><br>Grid size 2.5 mm	EPID aS1000 PortalDosimetry	2%/2 mm<br><br>5% threshold, N/A norm.	Range: 90-100%	31 features among complexity metrics and machine characteristics	AdaBoost<br>RF<br>XGBoost	MAE <= 1%<br>Max AbsErr: 3.9% (AdaBoost), 3.5% (RF), 4.5% (XGBoost)
Li et al. [2] (2019)	303 VMAT plans	Gynecologic<br>H&N	Trilogy<br><br>Millennium 120 MLC	N/A	MatriXX with MultiCube phantom	2%/2 mm<br>80% TL<br><br>3%/2 mm<br>90% TL<br><br>3%/3 mm<br><br>10% threshold, global norm.	10% of plans with GPR<80% at 2%/2 mm<br><br>12% of plans with GPR<90% at 3%/2 mm	54 complexity metrics	PL<br>RF	2%/2 mm<br>PL MAE 5.10% (max AbsErr 18%)<br>60% sensitivity, 100% specificity<br>RF 100% sensitivity, 44% specificity<br><br>3%/2 mm<br>PL MAE 2.98% (max AbsErr 12%)<br>33% sensitivity, 100% specificity<br>RF 67% sensitivity, 71% specificity<br><br>3%/3 mm<br>PL MAE 1.83% (max AbsErr 8%)
Hirashima et al. [3] (2020)	1255 VMAT plans	Multiple	Vero4DRT, TrueBeam, Clinac iX<br><br>6 MV, 10 MV, WFF, FFF	Acuros XB, Collapsed cone<br><br>Grid size < 2.5 mm	ArcCHECK	5%/1 mm<br>90% TL<br><br>2%/2 mm<br>80% TL<br><br>3%/2 mm<br>90% TL<br><br>10% threshold, global norm.	18% of plans with GPR<90% at 5%/1 mm<br><br>10% of plans with GPR<80% at 2%/2 mm<br><br>14% of plans with GPR<90% at 3%/2 mm	24 complexity metrics and 4 clinical parameters<br><br>+851 dosiomic features – here we consider only the plan complexity model	XGBoost	5%/1 mm<br>2.9% MAE, 83% sensitivity, 84% specificity<br><br>2%/2 mm<br>4.6% MAE, 70% sensitivity, 68% specificity<br><br>3%/2 mm<br>3.1% MAE, 64% sensitivity, 82% specificity
Wall and fontenot [4] (2020)	500 VMAT plans	Multiple	4 dosimetrically matched machines (Elekta)<br><br>WFF, FFF<br><br>Agility MLC	N/A	MapCHECK2 with MapPHAN phantom	3%/3 mm<br><br>10% threshold, local norm.	Test set mean ± std: 89.39% ± 6.01%	241 features among complexity metrics and plan parameters	LR<br>Elastic Net<br>SVM<br>DT<br>RF<br>AdaBoost<br>GBDT<br>ANN	MAE<br>LR 4.29%<br>Elastic Net 4.17%<br>SVM 3.85% (max AbsErr 23.7%)<br>DT 4.14%<br>RF 3.98%<br>AdaBoost 3.98%<br>GBDT 3.94% (max AbsErr 17.8%)<br>ANN 4.01%

Kusunoki et al. [5] (2022)	356 VMAT plans	H&N	Trilogy<br><br>6 MV<br><br>Millennium 120 MLC	Acuros XB (11.0)<br><br>Grid size < 2.0 mm	Delta4 Phantom	2%/2 mm<br>99% lower control limit<br><br>10% threshold, global norm.	Above 95.2%	15 complexity metrics	Ridge<br>RF<br>SVM<br>Stacking	100% sensitivity, 75% specificity
Han et al [6] (2023)	201 VMAT plans	Pelvis<br>H&N	Elekta Synergy<br><br>6 MV<br><br>MLCi2TM	Algorithm N/A<br><br>Grid size 3.0 mm	ArcCHECK with SNC Patient	2%/2 mm<br>80% TL<br><br>3%/2 mm<br>90% TL<br><br>3%/3 mm<br>95% TL<br><br>10% threshold, N/A norm.	10% of plans with GPR<80% at 2%/2 mm<br><br>10% of plans with GPR<90% at 3%/2 mm<br><br>7% of plans with GPR<90% at 3%/3 mm	13 plan complexity metrics<br><br>+833 dosiomic features – here we consider only the plan complexity model	RF	2%/2 mm<br>83.3% sensitivity, 83.6% specificity<br><br>3%/2 mm<br>87.5% sensitivity, 71.7% specificity<br><br>3%/3 mm<br>84.5% sensitivity, 100% specificity
Zhu et al. [7] (2023)	1383 IMRT fields	Multiple	Halcyon<br><br>SX2 dual-layered MLC	AAA (15.6.06)<br><br>Grid size 2.5 mm	Detector N/A<br>PortalDosimetry	1%/1 mm, 2%/2 mm, 3%/2 mm<br><br>10% threshold, N/A norm.	Only 2% of plans with GPR<90% at 2%/2 mm	33 complexity metrics	GBDT<br>RF<br>PL	MAE between 1% and 2%<br>Errors increased with stricter criterion
Lambri et al. [8] (2023)	12469 VMAT arcs	Multiple	TrueBeam<br><br>Millennium 120 MLC, HD 120 MLC<br><br>6 MV, 10 MV, WFF, FFF	AAA, AXB (15.6)<br><br>Grid size < 3.0 mm	EPID aS1000, aS1200<br>PortalDosimetry	3%/1 mm<br>95% TL<br><br>10% threshold, global norm.	17% of arcs with GPR<95%	19 features among complexity metrics and plan parameters	XGBoost	2.33% MAE, 61% sensitivity, 90% specificity
Noblet et al. [9] (2024)	1767 VMAT arcs	Multiple	TrueBeam<br><br>HD 120 MLC	AAA (13.7 or 16.1)<br><br>Grid size N/A	EPID aS1200<br>PortalDosimetry	2%/2 mm<br>98.3% TL<br><br>10% threshold, local norm.	10% of arcs with GPR<98.3%	19 complexity metrics	MLP	52% sensitivity, 92% specificity

Present Study	2430 HyperArc arcs	Brain	Edge<br><br>6 MV, 10 MV, FFF<br><br>HD 120 MLC	Acuros XB (15)<br><br>Grid size 2.5 mm	EPID aS1200 PortalDosimetry	3%/1 mm 95% TL<br><br>10% threshold, global norm.	25% of arcs with GPR<95%	15 features among complexity metrics and plan parameters	RF	2.6% MAE (max AbsErr 15%), 93% sensitivity, 56% specificity

Abbreviations: AAA = Analytical Anisotropic Algorithm, AbsErr = Absolute Error, ANN = Artificial Neural Network, DT = Decision Tree, EPID = Electronic Portal Imaging Device, FFF = Flattening Filter Free, GBDT = Gradient Boosting Decision Tree, IMRT = Intensity Modulated Arc Therapy, LR = Linear Regression, MAE = Mean Absolute Error, MLC = Multi Leaf Collimator, MLP = Multi-Layer Perceptron, N/A: Not Applicable, PL = Poisson Lasso, RF = Random Forest, SVM = Support Vector Machine, TL = Tolerance Limit, VMAT = Volumetric Modulate Arc Therapy WFF = With Flattening Filter.

Section S1

Estimation of workload reduction if implementing the ML model in clinical practice. Estimated times are normalized by 100 plans.

Assumptions:

- If one arc fails, then the entire plan needs replanning
- Percentage of failures = 25% (according to this study's dataset); 25 out of 100 plans
- Sensitivity = 93%; 93% of true "fail" are flagged before measurement
- Specificity = 56%; 44% of true "pass" are falsely flagged
- Reoptimization = 10 min
- Replanning + measurement = 60 min
- SRS plans are always measured
- One reoptimization is enough to prevent a later replanning + measurement

Baseline workload = 25 failures × 60 min = **1500 min**.

With the model:

- True failures flagged before measurement = 25 failures × 0.93 = 23.25 plans; reoptimized (10 min each)
- Missed failures = 25 − 23.25 = 1.75 plans; require the 60 min replanning + measurement
- True negatives = 100 – 25 = 75 plans
- False positives ("pass" incorrectly flagged) = 75 × (1 − 0.56) = 33 plans; unnecessary 10 min reoptimization

Reoptimization total time = (23.25 + 33) × 10 = 56.25 × 10 = 562.5 min.

Replanning + measurement total time = 1.75 × 60 = 105 min.

Workload with model = 562.5 + 105 = **667.5 min**.

Workload reduction:

- Absolute saving = 1500 − 667.5 = 832.5 min
- Relative reduction = 832.5 / 1500 × 100 = **55.5%**

References

1. Lam, D.; Zhang, X.; Li, H.; Deshan, Y.; Schott, B.; Zhao, T.; Zhang, W.; Mutic, S.; Sun, B. Predicting Gamma Passing Rates for Portal Dosimetry-based IMRT QA Using Machine Learning. *Med. Phys.* **2019**, *46*, 4666–4675, doi:10.1002/mp.13752.
2. Li, J.; Wang, L.; Zhang, X.; Liu, L.; Li, J.; Chan, M.F.; Sui, J.; Yang, R. Machine Learning for Patient-Specific Quality Assurance of VMAT: Prediction and Classification Accuracy. *International Journal of Radiation Oncology*Biology*Physics* **2019**, *105*, 893–902, doi:10.1016/j.ijrobp.2019.07.049.
3. Hirashima, H.; Ono, T.; Nakamura, M.; Miyabe, Y.; Mukumoto, N.; Iramina, H.; Mizowaki, T. Improvement of Prediction and Classification Performance for Gamma Passing Rate by Using Plan Complexity and Dosiomics Features. *Radiotherapy and Oncology* **2020**, *153*, 250–257, doi:10.1016/j.radonc.2020.07.031.
4. Wall, P.D.H.; Fontenot, J.D. Application and Comparison of Machine Learning Models for Predicting Quality Assurance Outcomes in Radiation Therapy Treatment Planning. *Informatics in Medicine Unlocked* **2020**, *18*, 100292, doi:10.1016/j.imu.2020.100292.
5. Kusunoki, T.; Hatanaka, S.; Hariu, M.; Kusano, Y.; Yoshida, D.; Katoh, H.; Shimbo, M.; Takahashi, T. Evaluation of Prediction and Classification Performances in Different Machine Learning Models for Patient-specific Quality Assurance of Head-and-neck VMAT Plans. *Medical Physics* **2022**, *49*, 727–741, doi:10.1002/mp.15393.

6. Han, C.; Zhang, J.; Yu, B.; Zheng, H.; Wu, Y.; Lin, Z.; Ning, B.; Yi, J.; Xie, C.; Jin, X. Integrating Plan Complexity and Dosiomics Features with Deep Learning in Patient-Specific Quality Assurance for Volumetric Modulated Arc Therapy. *Radiat Oncol* **2023**, *18*, 116, doi:10.1186/s13014-023-02311-7.
7. Zhu, H.; Zhu, Q.; Wang, Z.; Yang, B.; Zhang, W.; Qiu, J. Patient-specific Quality Assurance Prediction Models Based on Machine Learning for Novel Dual-layered MLC Linac. *Medical Physics* **2023**, *50*, 1205–1214, doi:10.1002/mp.16091.
8. Lambri, N.; Hernandez, V.; Sáez, J.; Pelizzoli, M.; Parabicoli, S.; Tomatis, S.; Loiacono, D.; Scorsetti, M.; Mancosu, P. Multicentric Evaluation of a Machine Learning Model to Streamline the Radiotherapy Patient Specific Quality Assurance Process. *Physica Medica* **2023**, *110*, 102593, doi:10.1016/j.ejmp.2023.102593.
9. Noblet, C.; Maunet, M.; Duthy, M.; Coste, F.; Moreau, M. A TPS Integrated Machine Learning Tool for Predicting Patient-Specific Quality Assurance Outcomes in Volumetric-Modulated Arc Therapy. *Physica Medica* **2024**, *118*, 103208, doi:10.1016/j.ejmp.2024.103208.
